# Multilingual Voice AI for Postoperative Cataract Follow-Up in Turkish Speaking Patients in the United Kingdom: Patient and Public Involvement Focus Group Study

**DOI:** 10.2196/90809

**Published:** 2026-07-22

**Authors:** Mertcan Sevgi, Ariel Yuhan Ong, Katie Lean, Sian Rees, Ernest Lim, David Adrian Merle, Alexander C Day, Badrul Hussain, Pearse A Keane, Aisling Higham, Roxanne Crosby-Nwaobi

**Affiliations:** 1 Institute of Ophthalmology, University College London, 11-43 Bath Street, London, England, EC1V 9EL, United Kingdom, 44 020 7608 6800; 2 NIHR Moorfields Biomedical Research Centre, London, England, United Kingdom; 3 Department for Ophthalmology, University of Tübingen, Tübingen, Baden-Württemberg, Germany; 4 Health Innovation Oxford and Thames Valley, Oxford, England, United Kingdom; 5 Ufonia Limited, Oxford, England, United Kingdom; 6 Centre for Assuring Autonomy, University of York, York, England, United Kingdom; 7 Moorfields Eye Hospital NHS Foundation Trust, London, England, United Kingdom; 8 Oxford University Hospitals NHS Foundation Trust, Oxford, England, United Kingdom

**Keywords:** artificial intelligence, voice assistant, multilingual, health equity, language barriers, patient and public involvement, co-design, cataract surgery, telephone, postoperative follow-up

## Abstract

**Background:**

Conversational voice AI assistants can automate postoperative follow-up calls in high-volume, low-complexity pathways such as cataract surgery but may widen health inequalities if language access and inclusive design are not built in. This patient and public involvement focus group was conducted to inform the Turkish-language adaptation of Dora ahead of a forthcoming multilingual clinical trial at Moorfields Eye Hospital.

**Objective:**

This study aims to inform the Turkish-language adaptation of Dora by gathering input from Turkish speaking community contributors about their experiences with UK ophthalmic care, language-related barriers, and design requirements for an equitable voice AI.

**Methods:**

We conducted a 1-time, 2-hour patient and public involvement focus group with 7 Turkish speaking adults recruited via the Derman community charity. The session ran in 2 phases: contributors first discussed their experiences with UK ophthalmic care, then evaluated a prerecorded Turkish-language telephone call from a voice AI to a Turkish speaking volunteer. The session was delivered bilingually, recorded with consent, and synthesized using an approach informed by the principles of reflexive thematic analysis. The voice AI uses automatic speech recognition and neural text-to-speech, with a large language model–based dialog manager for open-ended conversation within a postoperative review protocol.

**Results:**

Contributors described how pathway delays and limited language support shape their care, including reliance on family members for translation and concerns about privacy and autonomy. A language-concordant voice AI was conditionally acceptable for standardized postoperative follow-up, provided specific safeguards were met. Priorities included advance notice of calls, caller verification, privacy assurances, a clear standard Turkish accent at a slower pace, tolerance for regional dialects, interpersonal warmth, interactivity, accessibility for low vision and low literacy, and clinician escalation for complex issues. These priorities were synthesized into a 10-point checklist: preparation, verification, confidentiality, clarity and pace, voice, empathy, interactivity, dialect handling, accessibility, and efficiency.

**Conclusions:**

For patients facing language barriers, conversational voice AI may complement existing services when implemented with clear verification, privacy protections, and a defined scope under clinician oversight. The 10-item checklist will guide the Turkish-language adaptation of Dora and will be tested alongside similar consultations with other language communities in the forthcoming multilingual cataract follow-up trial.

## Introduction

The drive to enhance health care efficiency through digital innovation is a core tenet of the National Health Service (NHS) Long Term Plan. This has paved the way for advanced technologies like conversational AI [1]. A prime application for this technology is in postoperative follow-up calls in high-volume, low-complexity pathways such as cataract surgery, which is the most prevalent surgical procedure performed in the United Kingdom and across Europe today [2,3]. Autonomous clinical assistants, such as AI agents that conduct natural language phone conversations with patients, can automate this routine care. However, the market remains nascent, with only a small number of providers. Technologies such as Dora (Ufonia Ltd) offer a solution that is both scalable for clinicians and highly accessible for patients, requiring no specialist applications or devices [4]. Initial evidence from English language deployments shows these AI agents can perform follow-up assessments with clinical accuracy and safety comparable to human ophthalmologists [5], while achieving high usability and patient acceptability [4,6].

Despite considerable progress in innovative AI solutions, there have been concerns that they can widen health inequalities [7,8]. In the United Kingdom, there are 5.1 million people (close to 8% of the population) whose main language is not English [9]. For this population, language barriers are a well-documented clinical risk factor, linked to a higher incidence of misdiagnosis, medication errors, and poorer health outcomes [10,11]. National NHS guidance explicitly warns that digital tools may unintentionally perpetuate or even amplify health inequalities if language access and inclusive design are not core considerations [12]. Importantly, this issue extends beyond the United Kingdom. A systematic review synthesizing studies from 8 countries shows that language discordance reliably erodes communication, patient safety, and care quality, with unevenly available interpreter services adding operational burden. While general translation apps are sometimes used, their coverage and evidence base are limited [13]. Where interpreter services are available, professional medical interpreters reduce errors and improve comprehension, usage, clinical outcomes, and satisfaction. They often approach language-concordant care and outperform ad hoc interpreting; however, access remains inconsistent and can lengthen or add costs to visits [13,14].

The development of trusted and effective user-centered digital health technologies for diverse communities requires a foundational commitment to patient and public involvement (PPI). This principle is not merely best practice but a core requirement within UK regulatory and evidence frameworks, which mandate proof of user acceptability to ensure new tools are relevant and trustworthy [15]. By listening to the voices of patients and communities, we are creating inclusive services and mitigating systemic bias [16,17]. Our research operationalizes this guidance, applying a PPI methodology to the development of a multilingual AI assistant, rooting our work in the philosophy that research must be conducted “with” or “by” the public to leverage their “lived experience” [18]. This consultation addresses 1 element of that broader challenge: eliciting community-generated requirements to inform the Turkish-language adaptation of Dora ahead of the forthcoming multilingual trial.

This consultation reports on the foundational PPI activity for a forthcoming 10-language clinical trial of the conversational AI assistant Dora at the Moorfields Eye Hospital NHS Foundation Trust, which serves a sociodemographically diverse population in London, United Kingdom. We began our work by engaging with an often-underserved group: Turkish migrants living in London, whose insights are particularly valuable for designing voice-first technology accessible to users with varying levels of literacy. Turkish is among the 10 main non-English languages in England and Wales with the lowest proportion of speakers who report speaking English “well” or “very well” (69.9%), leaving almost 1 in 3 who cannot speak English “well” or “at all” [19]. This makes Turkish speaking patients particularly vulnerable to language-related barriers in health care and motivates our focus on this community as an exemplar for multilingual voice-AI design. In this consultation, we draw on community contributors’ input on the care pathway and on a prototype Turkish-language AI tool to inform the design of the multilingual adaptation.

This PPI consultation had a single overarching aim to inform the Turkish-language adaptation of Dora ahead of the forthcoming multilingual trial. This aim was pursued through three linked objectives: (1) to gather contextual input from Turkish speaking community contributors on their experiences of UK ophthalmic care relevant to a follow-up call, (2) to identify language-related barriers that the design of a voice-AI tool would need to address, and (3) to elicit community-generated requirements and concerns for the Turkish-language adaptation.

## Methods

### Design and Setting

We conducted a PPI focus group consultation lasting 2 hours. The session was held in May 2025, at the Education Centre of Moorfields Eye Hospital in London, providing a familiar and relevant clinical setting.

### Contributors and Recruitment

Community contributors were recruited through Derman, a charity providing health and social care support to the Kurdish-Turkish community [20]. Seven Turkish speaking adults (5 women and 2 men), including 2 married couples, took part. The group comprised individuals who had received a range of ophthalmic services and treatments for various conditions, such as preoperative and postoperative care for cataracts, management of diabetic retinopathy, surgical treatment for squint, and administration of intravitreal antivascular endothelial growth factor injections. One contributor was the parent-carer of children with an inherited retinal disease. All contributors had emigrated from Turkey and self-reported limited English proficiency. Contributors were compensated for their time and travel in line with the National Institute for Health and Care Research guidance for PPI and engagement [21]. During recruitment and consent, we explained that the session would have two parts: first, to gather contributors’ experiences of eye care; and second, to introduce a prototype technology we are developing and invite their insights and thoughts about it. We invited contributors with firsthand experience of the UK ophthalmic care pathway as Turkish speakers via Derman’s existing trusted community relationships to maximize reach within a seldom-heard group. Inclusion criteria were adults aged 18 years or older who self-identified as Turkishspeaking and who had accessed ophthalmic services in the United Kingdom; the only exclusion criterion was insufficient capacity to give informed consent. A sample of 7 was selected to balance depth of discussion with the logistical feasibility of a single 2-hour focus group, consistent with PPI practice for early-phase inclusive design activities.

### Focus Group Procedure

The session was facilitated to create a supportive and bilingual environment. The lead moderator (MS) is a clinician and AI researcher fluent in both Turkish and English. He was supported by Nurullah Turan from Derman, who served as a neutral interpreter. To ensure comprehensive data capture, RCN (the project’s PPI cosupervisor) acted as a cofacilitator, while KL (Senior Program Manager, Health Innovation Oxford and Thames Valley) took detailed field notes. Questions were posed in English and interpreted into Turkish, with contributors’ responses translated back into English by Nurullah Turan for the benefit of the full project team. The bilingual fluency of MS allowed real-time verification of interpretation fidelity in both directions. Questioning in English with interpretation (rather than wholly in Turkish) was a deliberate choice to enable real-time participation by the full multidisciplinary team and to preserve the exact bilingual fidelity of the topic guide’s technical content on AI; contributors’ own contributions were made in Turkish. MS had prior experience facilitating focus groups on conversational voice AI and, together with Nurullah Turan and RCN, held a presession briefing on power-dynamics mitigation: contributors were explicitly invited to disagree, critique, or raise concerns, and the presence of Nurullah Turan as a trusted community member was intended to reduce social-desirability bias. The session lasted approximately 2 hours, inclusive of a short break.

The focus group was structured into 2 distinct parts, following a semistructured interview topic guide (Multimedia Appendix 1). This guide was developed through an iterative process of discussion and review by the authors. The final questions reflect the team’s combined expertise in conducting focus groups on conversational AI and in applying PPI methodologies to promote health equity among underserved communities.

The initial phase of the session aimed to explore contributors’ general experiences with ophthalmic services in the United Kingdom. Topics included positive and negative aspects of their care, challenges related to language barriers, and their experiences with human interpreters as well as any previous automated telephone systems. This provided context for the second part of the session.

The second phase focused on eliciting reactions to the Dora prototype. Contributors listened to a prerecorded audio sample of a postoperative cataract follow-up call between Dora, speaking in Turkish, and a Turkish speaking volunteer. Their immediate, unprompted reactions to the voice and style of the call were then gathered. After their initial thoughts were shared, the moderator revealed that the call was conducted by an AI assistant. This prompted a broader discussion about trust, safety, potential benefits, and concerns regarding such technologies. Following this discussion, contributors were asked to listen to the recording a second time, with the specific instruction to focus critically on the linguistic and paralinguistic details of the AI’s speech, including its tone, pace, accent, and clarity. This 2-step process allowed us to capture both an initial holistic impression and a subsequent, more detailed technical critique.

With consent from all contributors, the entire session was audio recorded. Detailed field notes by KL captured nonverbal cues, group dynamics, and contextual observations that supplemented the audio recording.

### Technology: Dora

**Figure 1.**
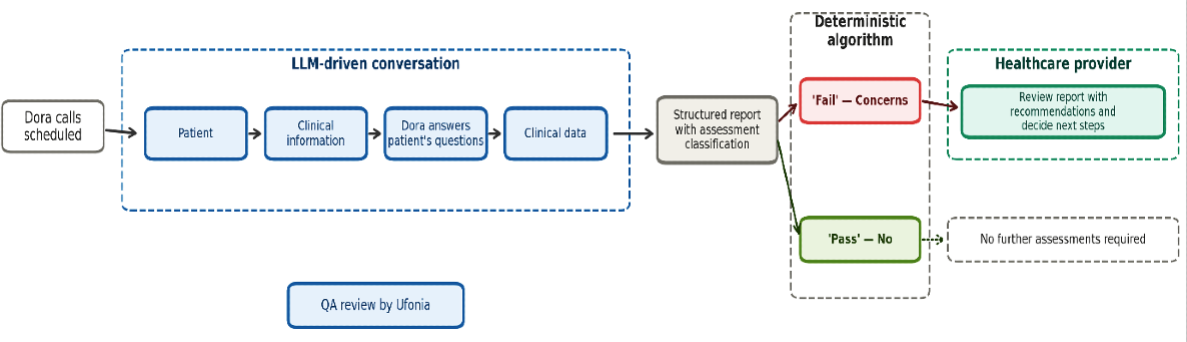
AI-assisted postoperative follow-up workflow (Dora). The system places an outbound call to the scheduled patient and conducts a large language model (LLM)–driven conversation: authentication, clinical information gathering, answering patient questions, and clinical data summarization. A deterministic algorithm generates a structured report and classifies the assessment as “Pass” (no concerns) or “Fail” (concerns identified). Clinicians review the report and determine the next steps. Quality assurance (QA) review by Ufonia Ltd provides additional oversight of conversation quality and safety. Shown here is the assistant that is the subject of the forthcoming multilingual clinical trial at Moorfields Eye Hospital and of the patient and public involvement consultation reported in this paper.

Dora is a UK Conformity Assessed class I software as a Medical Device used for autonomous, structured telephone conversations, such as follow-up after uncomplicated adult cataract surgery, typically 3 to 4 weeks postoperatively. The system runs as a cloud-hosted service and reaches patients via telephone, either on mobile or landline. In current English language NHS deployments, Dora conducts a protocolized postoperative conversation using a rule-based dialog engine to elicit a prespecified set of symptoms (redness, pain, visual issues, flashing lights, and floaters). A deterministic algorithm then generates a structured triage summary and a Pass or Fail recommendation for clinician review, with a structured report available to the care team [5]. For the forthcoming multilingual clinical trial, the conversational component will use a large language model (LLM) to support open-ended dialog in multiple languages while retaining the same deterministic triage step and clinician oversight (Figure 1).

### Analysis

Consultation input was synthesized using a structured approach informed by the principles of reflexive thematic analysis [22]. MS reviewed the audio recordings and field notes iteratively to identify recurring requirements, concerns, and design considerations raised by contributors. Candidate domains were reviewed and refined with RCN and KL. As is appropriate for a formative PPI consultation rather than a qualitative research study, we did not seek thematic saturation, and the synthesis was undertaken to surface design-relevant input rather than to produce generalizable findings about Turkish speaking patients. The consultation workflow is summarized in Textbox 1. This consultation is reported in accordance with the Guidance for Reporting Involvement of Patients and Public (GRIPP2) short-form checklist for reporting public involvement in research [23], the recognized reporting standard for PPI activities, with the completed checklist provided (Checklist 1). Our analytical approach was further informed by updated guidance from Braun and Clarke [24] on reflexive thematic analysis in health research; we acknowledge that the position of MS as a Turkish speaking clinician and AI researcher shaped both rapport and interpretation, and this is named here as part of routine analyst reflexivity for a PPI consultation, alongside cross-checking of the bilingual analyst’s reading of the audio against contemporaneous field notes of KL, and iterative team discussion of candidate domains.

Textbox 1. **Consultation methods overview: workflow summary.**

**Recruitment and contributors**

-  Community partner: Derman
-  Turkish speaking adults involved as patient and public involvement (PPI) contributors
-  National Institute for Health and Care Research–aligned compensation

**Session and procedure**

-  A 2-h PPI focus group at Moorfields Eye Hospital conducted bilingually
-  Part 1: personal experiences of the eye care pathway
-  Part 2: impressions of prototype Turkish voice AI and invited discussion
-  Bilingual moderation + interpreter support

**Data and analysis**

-  Audio recording
-  Field notes
-  Thematic synthesis informed by reflexive thematic analysis principles, conducted by a bilingual clinician-researcher with team review

**Outputs**

-  Themes 1-4 (conditional acceptance) of voice AI
-  Contributor‑derived 10‑point “What makes a good call” checklist
-  Inputs to planned 10-language clinical trial

### Ethical Considerations

This activity was classified as PPI using the UK Health Research Authority’s “Is my study research?” online decision tool [25], on the grounds that the activity did not aim to generate generalizable new knowledge, did not involve allocation to interventions for the purpose of evaluation, and sought contributions from community members in their role as public contributors rather than as research participants. As such, it did not require formal review by a research ethics committee. The completed output from the Health Research Authority decision tool is provided as Multimedia Appendix 2. In line with best practices for PPI, verbal consent was obtained from all contributors before the focus group began. Contributors were informed of the consultation’s purpose and their right to withdraw at any point before the analysis of the data. To protect contributor anonymity, all data are presented in an aggregated, nonidentifiable format.

## Results

**Figure 2.**
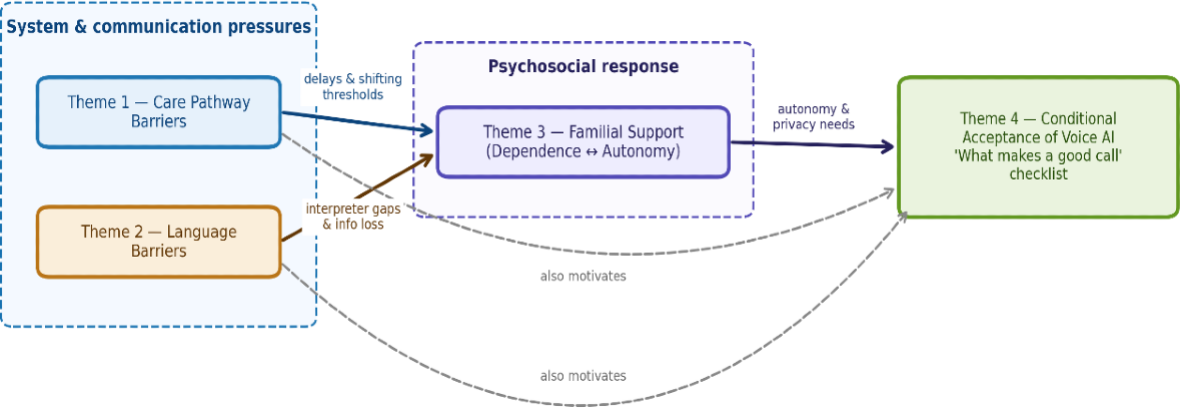
Thematic map of contributor perspectives. Themes derived from the thematic synthesis of contributors’ input are shown within grouping boxes. Theme 1 (care pathway barriers) and theme 2 (language barriers; system and communication pressures) feed into theme 3 (familial support: dependence ↔ autonomy; psychosocial response). These factors collectively lead to theme 4 (conditional acceptance of voice AI: “What makes a good call” checklist). The map derived from the 2-hour patient and public involvement focus group conducted with 7 Turkish speaking adults at Moorfields Eye Hospital, London, in May 2025, to inform the Turkish-language adaptation of a postoperative cataract follow-up voice–AI assistant.

Our analysis identified 4 primary themes that encapsulate the contributors’ experiences (Figure 2). These themes progress from systemic challenges in accessing care and communication to the resulting psychosocial dynamics and, finally, to a collective, pragmatic assessment of a potential technological solution.

### Theme 1: Barriers in the Care Pathway

A central theme was a perceived disconnect from the UK health care system, characterized by significant delays in receiving care and what contributors interpreted as divergent philosophies of care. Most of the contributors described being advised by their health care providers to wait for their conditions to worsen significantly before intervention was offered. This was framed as clinicians “shifting the threshold” for treatment, with statements like being told by their health care providers to “wait until it is really bad” for surgery. One contributor reported that they were informed by their health care provider that cataract surgery could be deferred until the age of 75 years, a recommendation that generated considerable concern among the focus group contributors.

This experience of prolonged waiting and perceived clinical passivity prompted 4 contributors to engage in transnational care-seeking, traveling to Turkey for more timely procedures. The contrast was starkly articulated by 1 individual: “Here they told me to wait; in Istanbul, they operated the same week.” This appeared to reflect a perceived mismatch in clinical thresholds between the 2 systems, which prompted some contributors to seek care abroad. These predominant narratives of frustration were punctuated by a small number of isolated positive encounters, such as proactive postoperative calls from a surgeon in the United Kingdom, highlighting that supportive care, while valued, was not the norm.

### Theme 2: Impact of Language Barriers

Contributors’ difficulties were shaped by language barriers, which created clinical risks and emotional distress. Interpreting services, typically delivered via telephone or in-person within NHS clinical settings, represent an essential support mechanism but were described by contributors as inconsistent and, at times, distressing. Contributors recounted experiences with interpreters who arrived late, behaved confrontationally, or terminated calls abruptly. This unreliability was compounded by a perceived lack of respect, with several contributors feeling that interpreters spoke “down” to them or omitted key clinical details. These gaps in communication led to tangible consequences, such as confusion over advice regarding driving at night after cataract surgery or fasting instructions before an endoscopy. To navigate these challenges, contributors developed workarounds, predominantly relying on their adult children for on-the-spot telephone translation or using digital tools like Google Translate.

### Theme 3: Navigating Familial Support

The reliance on family members as linguistic and logistical mediators revealed a tension between dependence and the desire for personal autonomy. While rooted in family solidarity, this reliance was frequently a source of interpersonal strain. Contributors described how requests for assistance could lead to frustration for both parties, with support often coming after “a thousand complaints,” generating feelings of guilt. This dynamic was reported to be growing more complex as adult children establish their own households and careers, diminishing their availability. As 1 contributor observed, “When they were younger they helped; now they say, ‘Sort it out yourself, use an interpreter.’”

The consequence of this evolving and often fraught dependence is a pronounced need for confidential and private communication regarding their health. Many contributors expressed a preference to discuss health matters without relatives or even official interpreters present, particularly for sensitive topics. The sentiment, “Sometimes I don’t want to tell the interpreter; I would rather speak in private,” underscores a powerful desire for an autonomous space to manage their health care. Together, the information gaps described in theme 2 and the strained reliance on family mediation described in theme 3 frame the specific affordances that contributors looked for in a voice-AI assistant, setting the agenda for theme 4.

### Theme 4: Conditional Acceptance of Voice AI

The challenges outlined in the preceding themes directly informed the group’s reaction to the prospect of an AI assistant. This generated a conditional acceptance of the technology, which contributors viewed as a potential solution to long-standing issues. The perceived benefits were directly linked to the problems of dependence and lack of privacy detailed in themes 2 and 3. For instance, an automated service in their own language was seen as a mechanism to increase patient autonomy and provide a confidential channel for health care communication, directly addressing the expressed desire to “speak in private” without family or interpreters present. In this consultation, Dora is delivered as an automated postoperative follow-up call, which is the same channel in which the communication problems described above recur; contributors’ feedback, therefore, applies directly to that channel.

However, this potential acceptance was dependent on several important considerations related to trust, safety, and the technology’s scope of practice, which the group offered as guiding principles for its development. Contributors’ skepticism toward unsolicited automated calls was clear, with 1 stating, “Usually when it’s a machine and it’s in English, I just put the phone down.” This sentiment, along with an awareness of fraudulent scams, highlighted the need for strong verification measures.

Consequently, several key suggestions were put forward to build trust. Contributors felt it was important that the service be officially introduced by their health care provider to establish legitimacy. They suggested that trust could be further established through practical verification measures. Suggestions for this included the AI stating the patient’s name and hospital number, or even providing preshared “pin codes for access” to prove the call was authentic. The group also recommended a clear boundary for the AI’s role, considering it appropriate for routine, informational tasks only and not for complex or emotionally sensitive consultations. Finally, they emphasized the need for explicit assurances of data confidentiality, expressing a strong preference that their information not be shared with “anyone other than our doctors.”

To translate the qualitative findings into actionable design principles, a portion of the focus group was dedicated to gathering contributors’ feedback on the prerecorded Dora call. Their responses, along with ideas that emerged organically throughout the discussion, were analyzed by the team. The key user requirements identified through this process were then synthesized into the following 10-point checklist, which frames the community’s essential criteria for a “good call” (Table 1). We use “standard Istanbul Turkish” here in the sense that “Received Pronunciation” or “Standard Southern British English” is used in English: the broadly understood, broadcast-standard variety as opposed to a regional dialect.

**Table 1.** “What Makes a Good Call?”: patient and public involvement (PPI)–derived checklist guiding multilingual voice–AI development^a^.

Principle	Community requirements or rationale and illustrative quote
Preparation	Advance warning or notification from their health care provider before the call. “Nobody told me not to eat. That’s why I had to go twice, not just once.”
Verification	A mechanism to prove the call is legitimate, such as the AI stating the patient’s name and hospital number to confirm its authenticity. “If the machine gives me my hospital number, then I’ll speak.”
Confidentiality	An explicit assurance that the conversation is private, and the information is shared only with their clinical team. “I wouldn’t want it shared with anyone other than the doctor.”
Clarity and pace	Speech should be clear, use a standard Istanbul Turkish accent, and be delivered at a slower, more deliberate pace. “It felt a bit fast. When it’s too fast you can lose something.”
Voice	A natural, human-sounding voice, with a choice of male or female tone. “Even if the other side isn’t actually a person, if it’s like you’re speaking with a human, you can give your answers better.”
Empathy	Use of warm, empathic phrasing throughout the conversation. “If the machine were always empathic and understood everyone very well, that would be good.”
Interactivity	A 2-way system that allows users to ask follow-up questions. “It was good, but after the second question, can you ask another question?”
Dialect handling	Robust enough to understand regional dialects while maintaining its own clarity. “The more languages it knows the better. But mother tongue is still something else.”
Accessibility	A voice-first interface suitable for users with low vision or limited literacy. “I can’t read or write. Because I have problems with my eyes, it’s easier for me to press, speak, and send.”
Efficiency	An evident time-saving benefit for routine tasks (eg, postoperative checks and medication guidance). “I find it very hard to go outside. After a hospital visit I sometimes don’t leave the house for months.”

a Through a 2-part session (experiences of eye care; introduction to a Turkish prototype), contributors articulated criteria they considered essential for trustworthy automated follow-up. The 10 principles cover precall set-up (Preparation, Verification), privacy and communication quality (Confidentiality, Clarity and Pace, Voice, Empathy), conversational function (Interactivity, Dialect Handling), and practical usability (Accessibility, Efficiency). The checklist is not rank-ordered; examples are indicative and will be iteratively validated with additional language communities. Derived from a single, 2-hour PPI focus group held at Moorfields Eye Hospital, London, in May 2025, with 7 Turkish speaking adults recruited via the Derman community charity, the session aimed to inform the Turkish-language adaptation of a postoperative cataract follow-up voice-AI assistant.

## Discussion

### Principal Findings

This PPI consultation produced a community-informed set of design requirements for the Turkish-language adaptation of Dora, which will feed directly into the forthcoming multilingual trial at Moorfields. The focus group discussion provided valuable insights into how this community assesses new health technologies. It became clear that contributors evaluated the AI assistant not as an isolated tool but in the context of their broader experiences navigating care pathways in the United Kingdom. Contributors’ input pointed to a recurring pattern in which pathway delays and language barriers led to family reliance, which in turn raised concerns about privacy and autonomy. These concerns then directly shaped the design requirements they articulated for a voice-AI tool: a language-concordant, identity-verifying, confidential channel that does not require family mediation.

This context is key, as it highlights why this patient group has so much to gain from a well-designed, language-concordant tool. While an automated assistant might offer convenience and efficiency for the general patient population, for contributors in this group, its value proposition is far more fundamental. The technology holds the potential to directly address and help dissolve some of the most persistent barriers they face. A system that can speak their language could mitigate the unreliability of interpreters, lessen the burden on family members, and restore a sense of privacy and independence in managing their health (theme 4).

This potential explains the conditional nature of the group’s enthusiasm, detailed in theme 4, where pragmatic concerns about security and data confidentiality were integral to their overall assessment. The “good call” checklist is the tangible output of this unified perspective. This checklist should be seen not as a list of demands but as a constructive and proactive set of design principles offered by the community to help ensure the technology successfully meets their distinct and significant needs. It provides a practical framework for developers to build a tool that addresses both the community’s desire for greater autonomy and their fundamental requirement for trust.

### Relevance to the Wider Literature on Voice AI and Language Access

Outbound telephony for follow-up spans a continuum from interactive voice response to conversational voice AI. Interactive voice response uses scripted, menu-driven prompts with keypad or simple speech recognition and has shown that the voice channel can reach patients with limited literacy, engage basic-phone users, and produce modest but meaningful improvements in engagement and some health behaviors in diverse settings, including low-resource contexts [26,27]. Our consultation builds on this infrastructure but examines a richer conversational voice AI that uses LLMs to support open-ended 2-way conversation in the patient’s preferred language. As a nascent technology, there is a limited amount of literature specifically exploring its use for outbound patient communication. The existing research landscape predominantly focuses on either clinician-facing AI (such as diagnostic tools) or patient-facing, text-based chatbots [28,29]. While our consultation echoes common themes found across this broader literature, such as the centrality of patient trust and concerns about privacy [30], its novelty lies in examining these issues within the unique context of spoken, automated interactions. Previous research has highlighted public and patient concerns regarding the lack of perceived empathy and the risk of depersonalization when using automated systems in health care [29,31]. Our findings align with this literature, as contributors in our focus groups articulated these same anxieties. While acknowledging the potential benefits of an AI-driven service, they expressed a clear preference for interacting with a voice that was not only human-like but also emotionally receptive. This expressed patient need for empathic interaction coincides with significant technological advancements in this domain. Recent evaluations of LLMs, particularly in health care contexts, have demonstrated that their responses can be comparable to, or even rated superior to, those of human clinicians in certain empathy-related tasks [32]. This development indicates that future iterations of voice AI may be capable of meeting the emotional and empathy standards required by patients, thereby addressing a key barrier to acceptance identified in both our consultation and the broader literature.

Our findings also intersect with literature on caregiver burden and language access. UK national guidance discourages reliance on family members for ad hoc interpretation because of risks to accuracy, confidentiality, and autonomy [33]. Systematic evidence shows that professional medical interpreters reduce errors and improve comprehension, usage, clinical outcomes, and satisfaction compared with ad hoc approaches, often approaching language-concordant care, although access can be inconsistent and resource-intensive [14]. Importantly, the access problem is international. A global systematic review spanning studies in both high-income and lower-resource settings reports that language barriers are linked to poorer safety and quality, that interpreter availability is frequently limited, and that when services are available, they may increase visit length and cost; the same review notes widespread real-world reliance on informal workarounds or basic translation tools with variable coverage [13]. We therefore position proactive, language-concordant voice AI as complementary to professional interpreting: suitable for standardized, low-risk outbound follow-up in the patient’s language, with clear escalation to clinicians and professional interpreters for complex or sensitive conversations. In this way, such tools may alleviate a discrete dimension of caregiver burden by restoring privacy and autonomy [34].

Finally, while English-language evaluations of Dora report safety, accuracy, and acceptability for cataract follow-up [4], the equity implications of multilingual deployment remain underexplored. Our community-derived checklist outlines trust, empathy, verification, privacy, and linguistic clarity for spoken, automated interactions, aligning with guidance that digital tools risk amplifying inequities if language access is not designed in from the outset [12]. This set of requirements will shape the Turkish-language adaptation of Dora, and related consultations are planned with other language communities included in the forthcoming multilingual trial.

Community contributors’ priorities resonate with constructs in the Technology Acceptance Model and the Unified Theory of Acceptance and Use of Technology [35,36], particularly performance expectancy, effort expectancy, social influence, and facilitating conditions, with Holden and Karsh noting the need to adapt these frameworks for health care contexts [37]. For language-minoritized users, linguistic access is a precondition for any of these constructs to be meaningfully assessed: a contributor whose Turkish the system cannot recognize cannot form a judgment about usefulness or ease of use at all. A formal evaluation against these frameworks is planned as part of the forthcoming multilingual clinical trial.

### Scope of This Consultation

This consultation drew on input from 7 Turkish speaking contributors recruited through a community organization. The contributors were, by definition, already connected to a community support network, so their input may not reflect the priorities of more isolated Turkish speakers—a gap that further consultation in the next phase of the design program will need to address. Their direct experience was with ophthalmic care, so the requirements they articulated for a follow-up tool will need to be tested with contributors from other clinical contexts as the multilingual program expands. As is appropriate for a formative PPI activity rather than a qualitative research study, we did not seek thematic saturation, and the consultation domains should be read as a community-generated starting point for design rather than as generalizable findings about Turkish speaking patients in the United Kingdom [38]. The scope was also intentionally limited to the patient perspective; further consultation with caregivers, family members, professional interpreters, and community organization staff is planned as part of the wider program. We worked directly from the bilingual audio recording and contemporaneous field notes rather than from a verbatim transcript; this prioritized pragmatic feasibility for a consultation activity over the linguistic depth that full bilingual transcription would allow.

### Implications and Next Steps

This PPI consultation suggests several key directions for the development of the multilingual Dora platform. The “good-call” checklist, derived from our work with the Turkish speaking community, provides an initial benchmark of patient-driven principles. Each PPI event with different language communities helps to iteratively shape and refine the core design of Dora. As such, these findings will be integrated with insights from our ongoing PPI work with other groups to ensure the final platform is grounded in a diverse range of patient perspectives. A critical part of this broader validation will involve formally assessing the system’s perceived empathy, cultural appropriateness, and language correctness, including technical error rates.

A key implication of our recruitment method is that proactive outreach will be essential in this next phase to identify and include “invisible” patients who are not connected to established community organizations, ensuring that the benefits of digital health tools are truly equitable.

Following this comprehensive PPI phase, the multilingual system, which is being developed and evaluated in 10 different languages, will be assessed in a clinical trial at a large tertiary eye care center serving a diverse population [39]. This trial will measure its impact on clinical safety, pathway efficiency, and health equity outcomes.

### Conclusions

This consultation provides insights from an underserved community, revealing how Turkish speaking patients perceive the role of voice AI in their health care. The contributors’ considerable optimism toward an automated telephone assistant did not stem from an affinity for technology but directly from the deeply felt challenges they face with language barriers, interpreter inconsistency, and the complex dynamics of relying on family for support. For a group that has contended with such obstacles, a well-designed AI tool is not seen as a convenience but as a tangible solution to problems of access, privacy, and autonomy.

The “good-call” checklist is a practical synthesis of this nuanced perspective, translating the principles of conditional acceptance from theme 4 into an actionable framework. By outlining the essential components of a successful automated call, from the linguistic quality of the AI’s voice to the critical principles for verification and confidentiality, the checklist provides guidance for developers. Taken together, this consultation reinforces that, for language-minoritized communities, trust, privacy, and inclusion are as central as translation itself to the design of digital health tools. By embracing meaningful PPI, the field can move beyond creating tools that are merely usable to developing AI that is genuinely empowering for all.

## Supplementary material

10.2196/90809 Multimedia Appendix 1 Topic guide. 1, 2, 3

10.2196/90809 Multimedia Appendix 2 Health Research Authority’s “Is my study research?” decision-tool output.

10.2196/90809 Checklist 1 GRIPP2 short-form checklist.

## Abbreviations

GRIPP2: Guidance for Reporting Involvement of Patients and Public
LLM: large language model
NHS: National Health Service
PPI: patient and public involvement
