# Supplementary material for: Multilingual Voice AI for Postoperative Cataract Follow-Up in Turkish Speaking Patients in the United Kingdom: Patient and Public Involvement Focus Group Study
Source: JMIR Form Res. 2026 Jul 22;10:e90809. doi: 10.2196/90809 (PMC13392653; doi:10.2196/90809)
Supplement: Multimedia Appendix 1 [file formative-v10-e90809-s001.docx]

## **Topic Guide**

### **Experiences of Eye Care**

**Aim:** To gather experiences of how people feel about the eye care they have received.

We would like to start by understanding a bit about your experience of receiving eye care. Can you share a bit about what it was like for you?

**Prompts**

- Thinking about your experience, what worked well for you or made it a good experience?
- When things have not gone so well, can you share a little about what was hard and what would have made it better?
- When you have been receiving eye care, has language ever been a challenge for you or your family?

#### **Interpreters**

Can you share your experience (if any) of when interpreters have been used to support your care?

**Prompts**

- What worked well?
- What do you think might have worked better?

#### **Health Care Method**

Have you ever received any automated telephone calls? (If time, branch out to not just health care.)

**Prompts**

- Can you share what that was like for you and what worked?
- What could have been done to make it better for you?

Have you ever received a telephone or video call for health care? Explore what worked and what did not.

**Additional question (if time)**

What one thing could doctors or nurses do to support clinical discussions with a member of the Turkish community?

### **Introduction of AI in Health Care and Dora**

**Aim:** To understand how people feel about receiving an audio recording from a conversational AI assistant. Gather thoughts on whether technology could be useful, nuances in translation, and explore trust, privacy, access to devices, etc.

We touched upon receiving automated telephone calls in the last session. Now we are going to play a short audio recording of a conversation between an artificial intelligence chatbot that has been trained to undertake conversations with patients following cataract surgery.

As you listen, we would like you to think about things such as:

- How it makes you feel
- How the language sounds
- The accent and speed of conversation

**Play the recording of Dora in Turkish**

Can we start by finding out your first reactions to the voice and style of the AI call?

**Prompts**

- How does it make you feel?
- Is the language correct (sound, accent, speed)?

How do you feel about the possible introduction of a telephone assistant like Dora?

**Prompts**

- In what way might it be helpful in your care?
- What might be good about using a technology like Dora?
- What concerns might you have about the introduction of technology like this?
- What would make you trust this sort of technology?
- How does it compare to other automated telephone assistants you might have used?
- What do you think others in your community might think?

**Prompts**

- What might stop someone from using it?
- What about access by phone or landline? Can everyone do this easily?
- Are there any changes you might make to make Dora more useful or trustworthy?
