# Supplementary material for: Multilingual Voice AI for Postoperative Cataract Follow-Up in Turkish Speaking Patients in the United Kingdom: Patient and Public Involvement Focus Group Study
Source: JMIR Form Res. 2026 Jul 22;10:e90809. doi: 10.2196/90809 (PMC13392653; doi:10.2196/90809)
Supplement: Checklist 1 [file formative-v10-e90809-s003.docx]

# GRIPP2 short-form checklist

Reporting checklist for this Patient and Public Involvement (PPI) consultation, completed in accordance with: Staniszewska S, Brett J, Simera I, et al. GRIPP2 reporting checklists: tools to improve reporting of patient and public involvement in research. *BMJ* 2017;358:j3453.

| **Section and topic** | **Item** | **How addressed in this consultation** |
| --- | --- | --- |
| **1: Aim** | Report the aim of PPI in this consultation. | To inform the Turkish-language adaptation of Dora ahead of the forthcoming multilingual trial at Moorfields Eye Hospital, by gathering Turkish-speaking community contributors’ input on their experiences of UK ophthalmic care, the language-related barriers they encounter, and their requirements for an equitable voice AI assistant.  Reported in: Abstract (Objective); Introduction (final paragraph). |
| **2: Methods** | Provide a clear description of the methods used for PPI in this consultation. | Seven Turkish-speaking community contributors (five women, two men, including two married couples) were recruited via Derman, a Kurdish-Turkish community charity in London. A single two-hour bilingual focus group was held in May 2025 at Moorfields Eye Hospital. The lead facilitator (MS) is a Turkish-speaking clinician and AI researcher; he was supported by a neutral interpreter from Derman (NT), a co-facilitator (RC-N), and a notetaker (KL).  The session had two phases: (i) contributors’ experiences of UK ophthalmic care; (ii) reactions to a pre-recorded prototype Turkish-language voice AI call (Dora). Input was audio-recorded with consent and synthesised using an approach informed by the principles of reflexive thematic analysis. Contributors were compensated in line with NIHR PPIE guidance.  Reported in: Abstract (Methods); Methods (Design & Setting; Contributors & Recruitment; Focus Group Procedure; Analysis); Ethical Considerations. |
| **3: Consultation results** | Outcomes: report the results of PPI in this consultation, including both positive and negative outcomes. | Contributors’ input pointed to a recurring pattern in which pathway delays and language barriers led to family reliance, which in turn raised concerns about privacy and autonomy. These concerns shaped the design requirements they articulated for a voice-AI tool: a language-concordant, identity-verifying, confidential channel that does not require family mediation.  Positive input: conditional acceptance of a language-concordant voice AI for standardised post-operative follow-up, with concrete priorities including a standard Istanbul Turkish accent with tolerance for regional dialects, slower pace, interpersonal warmth, genuine interactivity, and clear escalation pathways to clinicians. Negative input: scepticism towards unsolicited automated calls, concerns about caller verification and fraudulent scams, and discomfort with family mediation of sensitive health discussions.  These were synthesised into a 10-point design checklist (“What Makes a Good Call?”) covering preparation, verification, confidentiality, clarity and pace, voice qualities, empathy, interactivity, dialect handling, accessibility, and efficiency.  Reported in: Results (Themes 1 to 4; Table 1). |
| **4: Discussion and conclusions** | Outcomes: comment on the extent to which PPI influenced this consultation overall. Describe positive and negative effects. | Positive effects: contributors’ priorities have been incorporated directly into the technical specification and script development for the Turkish-language adaptation of Dora ahead of the multilingual trial, in particular the choice of a standard Istanbul Turkish accent, slower pace, dialect tolerance, identity-verification protocols, and explicit privacy assurances. The 10-point checklist serves as a community-informed benchmark that will guide parallel consultations with other language communities in the trial.  Negative effects: the consultation surfaced trade-offs that the wider design programme will need to navigate iteratively, including the boundary between automated triage and human clinician oversight, and the tension between household-level support structures and individuals’ desire for private, autonomous communication with healthcare services. These trade-offs were not fully resolved within a single consultation and remain open questions for the trial.  Reported in: Discussion (Principal Findings; Implications and Next Steps). |
| **5: Reflections / critical perspective** | Comment critically on this consultation, reflecting on the things that went well and those that did not, so others can learn from this experience. | What went well: recruitment via a single trusted community organisation (Derman) enabled rapid access to seldom-heard voices and supported open contribution from contributors with varying English proficiency. The bilingual session format, with NT serving as a neutral interpreter and MS providing real-time bilingual fluency, preserved the technical fidelity of the topic guide while keeping the multidisciplinary team engaged in real time. NIHR-aligned compensation and a familiar clinical setting (Moorfields Education Centre) supported informed and unhurried contribution.  What did not go as well: contributors were, by definition, already connected to community support, so their input may not reflect the priorities of more isolated Turkish speakers; further consultation in the next phase of the design programme will need to address this gap. The two-hour single session and bilingual interpretation imposed time pressure that constrained how deeply some topics could be probed. We worked directly from the audio recording and contemporaneous field notes rather than a verbatim transcript, prioritising pragmatic feasibility for a consultation activity over the linguistic depth that full bilingual transcription would allow. The lead facilitator’s dual position as a Turkish-speaking clinician and AI researcher shaped both rapport and interpretation; this was named openly and offset by iterative team discussion of candidate domains, but a fully independent analyst was not available within the scope of this consultation.  Reported in: Discussion (Scope of this consultation). |
